# From Detection to Decision: T-Score-Guided Use of STIR MRI After CT in Vertebral Fractures

**DOI:** 10.3390/diagnostics15182370

**Published:** 2025-09-18

**Authors:** Lior Yehuda Fitoussi, Árpád Viola, Siran Aslan, Réka Viola, Viktor Foglar, Mohammad Walid Al-Smadi

**Affiliations:** 1 Department of Neurotraumatology, Semmelweis University, 1081 Budapest, Hungarydrsiran.5@gmail.com (S.A.); foglar.viktor@gmail.com (V.F.); smadi996@hotmail.co.uk (M.W.A.-S.); 2 Department of Neurosurgery, Dr. Manninger Jenő Traumatology Institute, 1081 Budapest, Hungary; 3 Doctoral School of Clinical Medicine, Semmelweis University, 1083 Budapest, Hungary; 4 Department of Psychiatry, Peterfy Sandor Hospital, 1076 Budapest, Hungary; pallareka1004@gmail.com

**Keywords:** STIR MRI, CT, DEXA scan, T-score evaluation, osteoporotic fracture, spine fracture, diagnosis protocol, radiological assessment, osteoporosis

## Abstract

**Background/Objectives:** Vertebral fractures are frequently underdiagnosed after minor trauma in patients with normal or mildly reduced bone mineral density (BMD). CT, the standard first-line imaging, may miss subtle fractures, while STIR MRI is more sensitive but not routinely applied. We evaluated whether DEXA-derived T-scores can guide selective use of STIR MRI in patients > 50 years. **Methods:** We retrospectively analyzed 214 patients who underwent CT, sagittal whole-spine STIR MRI, and DEXA within 48 h after minor trauma. Fracture counts were compared using the Wilcoxon signed-rank test. Spearman’s correlation examined associations between T-score and fracture counts. Subgroups were defined as normal (≥−1), osteopenia (−2.5 < T-score < −1), osteoporosis (−3.5 < T-score ≤ −2.5), and high-risk osteoporosis (≤−3.5). **Results:** STIR MRI identified more fractures than CT in 87 patients (40.7%), while CT detected more in 19 (8.9%) (*p* < 0.0001). MRI outperformed CT across all T-score categories. The osteopenia group had the highest number of additional fractures (*n* = 53), and even patients with normal BMD showed a notable yield (*n* = 36). Correlations between T-score and fracture counts were weak and not statistically significant. **Conclusions:** T-score can support imaging triage but should not be used as a strict threshold. STIR MRI is justified in patients with T-scores < −2.5 when clinical suspicion exists and should be considered in those with higher T-scores if CT is negative but symptoms persist. Integrating T-score into imaging protocols may reduce missed fractures and improve outcomes.

## 1. Introduction

Vertebral fractures represent nearly one-third of all osteoporotic fractures worldwide and are strongly associated with increased morbidity, reduced quality of life, and higher mortality in older adults [1,2,3]. These fractures often result from low-energy trauma and are especially common in elderly populations with compromised bone integrity [2,4,5]. However, many vertebral fractures remain undiagnosed due to their subtle clinical presentation or complete lack of symptoms [2,3,4,5]. Missed fractures may lead to progressive spinal deformity, impaired mobility, and a heightened risk of subsequent fractures [6,7,8].

Imaging plays a central role in detecting vertebral fractures. CT is widely used in emergency settings due to its speed, accessibility, and ability to visualize bone architecture [9]. However, its sensitivity for early osteoporotic fractures—especially those without displacement—is limited [10,11,12,13,14,15]. In contrast, MRI, particularly using sagittal STIR sequences, can detect subtle marrow changes and trabecular disruption indicative of acute injury [16,17,18]. Despite this, MRI is not routinely used for all trauma patients due to limited availability, longer acquisition times, and higher costs.

This creates a critical diagnostic dilemma: when should clinicians escalate from CT to MRI in patients who present with minor trauma and have no overt signs of osteoporosis? While clinical judgment remains central, there is a need for objective criteria to guide imaging decisions. Bone mineral density (BMD), assessed via DEXA, offers a quantitative measure of fracture risk through the T-score, yet its role in imaging triage remains poorly defined. T-scores may provide an applicable threshold to identify patients in whom CT alone may be insufficient [19,20].

Despite advancements in imaging, the American College of Radiology highlights that inappropriate or delayed imaging strategies in spinal trauma contribute to missed diagnoses, prolonged hospitalization, and unnecessary healthcare expenditures [21]. A selective, T-score-guided approach could improve diagnostic accuracy and resource allocation without overburdening imaging systems.

This study investigates whether T-score thresholds can guide the decision to perform MRI following CT in patients over 50 with suspected vertebral fractures, by identifying subgroups in which MRI is likely to reveal additional injuries not visible on CT.

## 2. Materials and Methods

This retrospective observational study included patients who sustained minor spinal trauma between 1 July 2019 and 30 June 2023. From an initial cohort of 782 individuals diagnosed with thoracolumbar fractures, 214 patients were selected based on the availability of all three essential imaging modalities: computed tomography (CT), sagittal whole-spine short tau inversion recovery (STIR) magnetic resonance imaging (MRI), and dual-energy X-ray absorptiometry (DEXA).

Inclusion criteria:

- Age over 50 years.
- Minor spinal trauma.
- Thoracolumbar fracture confirmed by imaging.
- Availability of CT and MRI within 48 h of injury.
- DEXA performed within 48 h of injury. For each patient, both lumbar spine and hip T-scores were reviewed. The lower of the two values was used for classification.

Exclusion criteria:

- High-energy trauma.
- Incomplete imaging or missing T-score data.
- Examinations were performed outside the 48 h window of the injury.
- Poor image quality.
- Major discordance between spine and hip T-scores (>2 SD difference).

Fractures were identified through manual review of CT and MRI scans by two independent observers. The number of vertebral fractures was recorded for each patient across both imaging modalities. MRI was performed using sagittal STIR sequences.

Data were collected into Microsoft Excel spreadsheets. Spearman correlation coefficient was used to assess relationships between T-score values and fracture counts on both CT and MRI. Statistical analyses were performed using GraphPad Prism version 10 (GraphPad Software, San Diego, CA, USA). The Wilcoxon signed-rank test was used to compare fracture counts detected by CT and STIR MRI at both the overall cohort level and within T-score subgroups (normal, osteopenia, osteoporosis, high-risk osteoporosis). Holm–Bonferroni correction was applied to adjust for multiple subgroup comparisons. Effect sizes were described using the median MRI–CT difference per subgroup.

## 3. Results

### 3.1. Fracture Detection

MRI identified a total of 383 vertebral fractures, compared to 244 detected by CT, yielding a net difference of 139 additional fractures across the cohort (n = 214). On a per-patient basis, MRI revealed more fractures than CT in 87 patients (40.6%), while CT surpassed MRI in only 19 patients (8.9%). A paired analysis confirmed this difference was statistically significant (Wilcoxon signed-rank test, *p* < 0.0001).

Subgroup analyses also demonstrated statistically significant differences across all T-score categories after Holm–Bonferroni correction (Table 1). The median MRI–CT difference was 0 in the normal, osteopenia, and osteoporosis groups, reflecting that most patients had equal fracture counts on both modalities, but enough patients had additional MRI-only fractures to produce statistical significance. In the high-risk osteoporosis group, the median difference was 1, indicating a consistent added diagnostic yield of MRI in this subgroup.

### 3.2. T-Score Characteristics

#### 3.2.1. Overall T-Score Distribution and Gender-Based Risk Profile

The mean T-score was −1.8. Among the 214 patients, 42.5% were osteopenic (−2.5 < T-score < −1), 26.2% had normal bone density, 21.0% had osteoporosis (−3.5 < T-score < −2.5), and 10.3% had high-risk osteoporosis (T-score < −3.5). Of 143 females and 71 males, 48 females (33.6%) and 19 males (26.8%) were classified as having either osteoporosis or very low bone density osteoporosis (Table 1).

Among the study population, 71 males and 143 females were included. Of these, 19 males (26.8%) and 48 females (33.6%) were diagnosed with either osteoporosis or high-risk osteoporosis (High Risk population). This indicates a higher proportion of high-risk bone density classifications among female participants.

#### 3.2.2. Age-Stratified T-Score Distribution and Risk Classification

Patients aged 50–69 were most commonly classified as low risk (normal or osteopenia), comprising 74.3% and 78.3% of their respective age groups. In contrast, the proportion of high-risk diagnoses (osteoporosis and high-risk osteoporosis) increased with age, reaching 37.5% in the 70–79 group, 36.0% in the 80–89 group, and 14.3% in the 90–99 group. These findings reflect a clear age-related shift toward lower bone density, beginning most notably in the eighth decade of life.

The 70–79 age group consistently showed the highest prevalence across all T-score categories. Patients with normal bone density were most found in the 70–79 (35.7%) and 60–69 (26.8%) age ranges. Osteopenia peaked at 32.1% in those aged 70–79, with smaller but equal proportions in the 60–69 and 80–89 groups (23.1% each). Osteoporosis was also most frequent in the 70–79 group (42.2%), followed by 26.7% in the 80–89 group. Similarly, high-risk osteoporosis was concentrated in these same two groups, with 50% in 70–79 and 27.3% in 80–89 (Table 2).

### 3.3. Comparison and Correlation

#### 3.3.1. Correlation Between T-Score and Fracture Counts

**Figure 1:**
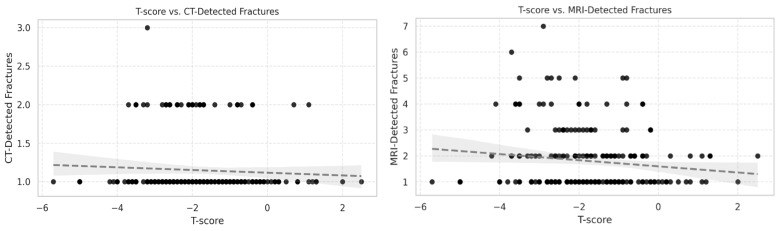
Scatter plots showing the relationship between T-score and vertebral fractures detected by (**left**) CT and (**right**) STIR MRI in 214 patients aged >50 years. Each point represents one patient. Correlations were weak and not statistically significant (CT: Spearman ρ = 0.08, 95% CI [−0.04 to 0.21], *p* = 0.23; MRI: ρ = −0.06, 95% CI [−0.19 to 0.07], *p* = 0.38). Data are widely dispersed, with no threshold effect observed.

No statistically significant correlations were found between T-score and the number of vertebral fractures detected by CT (Spearman ρ = 0.08, 95% CI [−0.04 to 0.21], *p* = 0.23) or STIR MRI (ρ = −0.06, 95% CI [−0.19 to 0.07], *p* = 0.38) (Figure 1). In both scatter plots, the data points were widely dispersed across the T-score range, with no clustering that would indicate a threshold effect. Although the scatterplots showed a weak inverse slope, this pattern was inconsistent and not statistically supported.

#### 3.3.2. Correlation Between T-Score and MRI–CT Fracture Difference

**Figure 2:**
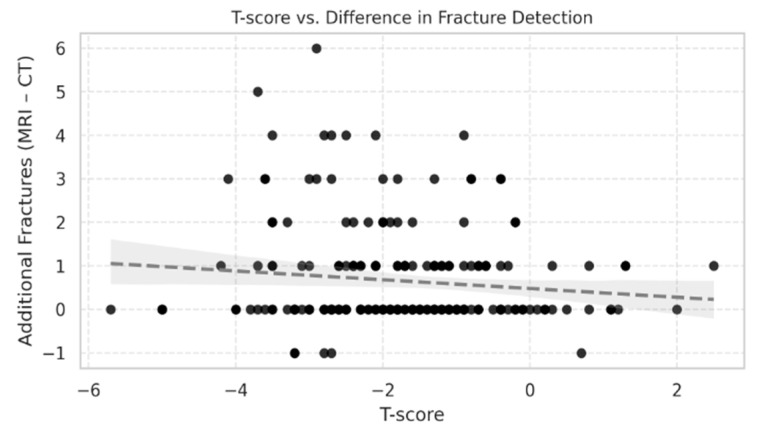
Scatter plot showing the relationship between T-score and the difference in fracture counts between STIR MRI and CT (MRI–CT) in 214 patients aged >50 years. Each point represents one patient. The correlation was weak and not statistically significant (Spearman ρ = −0.10, 95% CI [−0.24 to 0.04], *p* = 0.14). Data show wide variability, with additional MRI-detected fractures present across all T-score categories.

The difference in fracture counts between STIR MRI and CT (MRI–CT) also showed a weak, non-significant inverse correlation with T-score (Spearman ρ = −0.10, 95% CI [−0.24 to 0.04], *p* = 0.14) (Figure 2). The scatter plot displayed broad variability, with patients across all T-score categories showing both small and large positive differences. While a weak inverse slope was visible, the association was not statistically significant and had minimal explanatory value (R^2^ = 0.0128).

#### 3.3.3. Fracture Detection Discrepancy Between MRI and CT by T-Score Category

**Figure 3:**
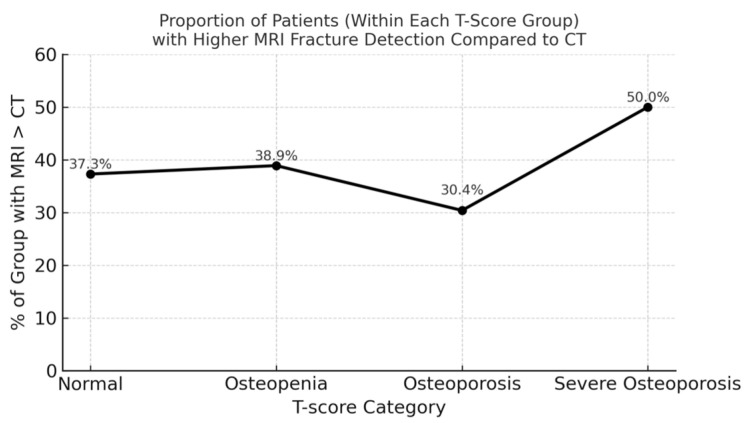
Additional vertebral fractures detected by STIR MRI compared with CT, stratified by T-score category. MRI identified more fractures in 22 patients with normal BMD (36 fractures), 37 with osteopenia (53 fractures), 14 with osteoporosis (36 fractures), and 11 with high-risk osteoporosis (26 fractures). The osteopenia group accounted for the greatest absolute number of MRI-only fractures, while the high-risk osteoporosis group showed the highest proportion of patients affected (50%). These findings indicate that clinically relevant diagnostic discrepancies occur across the full spectrum of bone mineral density.

When stratified by T-score category, MRI detected more fractures than CT in all four groups. Among patients with normal bone density (n = 59), MRI identified more fractures than CT in 22 individuals (37.3%), with a total of 36 additional fractures. In the osteopenia group (n = 95), 37 patients (38.9%) had more fractures on MRI than on CT, accounting for a total of 53 additional fractures. Among those with osteoporosis (n = 46), 14 patients (30.4%) showed additional MRI-detected fractures, with a cumulative difference of 36 fractures. Finally, in the high-risk density osteoporosis group (n = 22), 11 patients (50.0%) had more fractures on MRI than on CT, yielding 26 additional fractures (Figure 3).

The total cumulative fracture count difference (MRI minus CT) across categories was:

- Normal: 36 fractures (in 22 patients)
- Osteopenia: 53 fractures (in 37 patients)
- Osteoporosis: 36 fractures (in 14 patients)
- High-risk osteoporosis: 26 fractures (in 11 patients)

Although the proportion of patients with additional MRI findings was highest in the high-risk osteoporosis group (50.0%), the greatest number of additional fractures was observed in the osteopenia group (n = 53). This pattern persisted despite the lack of significant correlation between T-score and fracture count differences. These findings indicate that while both the frequency and magnitude of diagnostic discrepancies vary across bone density categories, they do not follow a linear trend with T-score. MRI thus provides consistent diagnostic advantages across the spectrum of bone mineral density, and the T-score alone is insufficient to predict the degree of mismatch between MRI and CT.

## 4. Discussion

This study highlights the value of integrating three diagnostic modalities—DEXA T-scores, CT, and STIR MRI—not only for assessing fracture risk but also for exploring whether T-score can serve as a practical decision-making tool for when to escalate from CT to STIR MRI in patients with suspected vertebral fractures. While the superior sensitivity of STIR MRI over CT for detecting vertebral fractures is well established [16,17,19,20,22,23], there is limited evidence on how quantitative bone mineral density measures might influence the diagnostic gap between these modalities [24]. By directly correlating T-scores with fracture detection differences, our study addresses a gap in the literature and takes a first step toward an evidence-based, T-score-informed imaging pathway.

### 4.1. Integration of T-Score, CT, and STIR MRI in Vertebral Fracture Assessment

Across all T-score categories, STIR MRI consistently identified more fractures than CT. The largest absolute number of additional fractures occurred in osteopenic patients, while the highest proportion of patients benefiting from STIR MRI was seen in high-risk osteoporosis. Importantly, even patients with normal bone density demonstrated a substantial yield from STIR MRI, reinforcing that occult vertebral fractures are not limited to those with advanced bone loss [17,22,23,25].

Although lower T-scores tended to correspond with higher fracture counts, correlation analyses were weak and not statistically significant, underscoring that T-score cannot serve as a predictive threshold on its own. Instead, it should be considered a supportive parameter that complements clinical findings and imaging, rather than a strict decision cut-off. This aligns with known pathophysiology, as lower bone density predisposes to subtle trabecular injuries detectable by MRI through marrow changes before structural collapse [17,20].

CT detected more fractures in approximately 9% of patients. These cases likely represent chronic or healed fractures without marrow edema, sclerotic deformities, or degenerative changes that are more conspicuous on CT due to its superior depiction of cortical and trabecular bone [11,14,21]. Such findings highlight the complementary roles of CT and MRI: CT remains indispensable for delineating chronic structural changes, while MRI is more sensitive for acute or occult injuries [14,15,21].

Subgroup-level paired analyses confirmed that MRI detected significantly more fractures than CT across all T-score categories, even after adjustment for multiple comparisons. However, the clinical interpretation requires caution: the median MRI–CT difference was 0 in the normal, osteopenia, and osteoporosis groups, suggesting that while MRI identified additional occult fractures in some patients, most individuals had concordant findings. In contrast, the high-risk osteoporosis subgroup showed a median MRI advantage of one fracture per patient, highlighting this group most consistently benefiting from STIR MRI escalation. These results reinforce that the T-score can inform imaging strategy, but correlations were weak, and no strict thresholds can be defined. T-score should therefore be viewed as a supportive parameter to prioritize MRI use in high-risk patients, while recognizing that clinically meaningful MRI-only fractures may also occur in normal and osteopenic patients.

Age-stratified analysis confirmed that osteoporosis and high-risk osteoporosis were most prevalent among patients ≥ 70 years [25,26]. However, the MRI–CT detection gap did not strictly follow an age gradient, supporting the view that bone density—not chronological age—is the more relevant factor for imaging triage. While osteoporosis was more common in women (33.6%), more than one-quarter of men (26.8%) in our cohort were also osteoporotic, underscoring the importance of screening across sexes.

### 4.2. Clinical Application of T-Score in STIR MRI Triage

From a clinical standpoint, T-score offers a pragmatic framework for guiding imaging escalation beyond CT in suspected vertebral fractures. In patients with T-scores below −2.5, fracture susceptibility is high due to structural bone weakness. STIR MRI is justified almost by default when there is any clinical suspicion—such as focal pain, tenderness, or symptoms disproportionate to CT findings—regardless of whether CT appears normal. In this high-risk group, the role of STIR MRI is well established, as these patients are vulnerable to both overt and occult fractures, and CT’s inability to depict early marrow changes may delay diagnosis and treatment.

**Figure 4:**
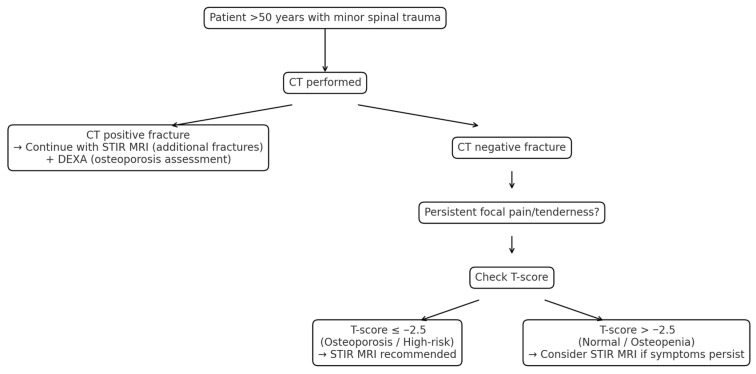
Decision pathway for patients > 50 years with minor spinal trauma. CT is first-line; if positive, protocol continues with STIR MRI for additional fractures and DEXA for osteoporosis assessment. If CT is negative, STIR MRI is guided by symptoms.

The more practice-changing implication of our findings lies in the T-score ≥ −2.5 group. In current clinical practice, negative CT findings in these patients often lead to termination of the imaging work-up. However, our correlation analyses did not reveal statistically significant associations strong enough to define a threshold between T-score and the extent of MRI–CT discrepancy. This indicates that the T-score should not be used as a strict threshold but as a supportive parameter that can complement clinical findings and imaging in guiding decision-making Figure 4.

The combined use of three modalities in this study is notable. Prior research has typically examined CT, STIR MRI, and DEXA separately—comparing CT to STIR MRI for fracture detection [17] or DEXA to CT for bone quality assessment [24,27]—but rarely correlates all three in the same cohort. By bridging this gap, our findings demonstrate how the T-score can be interpreted alongside CT and STIR MRI to guide when further imaging is warranted.

Incorporating such an approach into imaging appropriateness frameworks—such as the ACR Appropriateness Criteria [21,28]—could help standardize STIR MRI use in spinal trauma, particularly in complex cases with inconclusive CT findings or multifocal symptoms.

Regarding cost-effectiveness, STIR MRI requires additional resources, but sagittal sequences can typically be acquired within 5–10 min and provide early diagnostic information that prevents missed or delayed diagnoses. Avoiding undetected fractures may reduce downstream costs associated with hospitalization or surgical intervention, potentially offsetting the upfront imaging burden. Thus, while formal cost-effectiveness analysis was beyond the scope of this study, STIR MRI is likely to represent a clinically and economically justified adjunct in selected patients [16].

### 4.3. Strengths and Limitations

The main strength of this study lies in its multimodal design, which directly correlates DEXA T-scores, CT, and STIR MRI within the same cohort. By focusing on patients over 50 with minor trauma, it addresses a common but underexplored clinical scenario and highlights a real-world diagnostic gap. The systematic inclusion of all three modalities allowed us to assess fracture detection across bone density categories and evaluate whether the T-score can inform imaging triage.

Several limitations must be acknowledged. The retrospective, single-center design limits generalizability, and the inclusion of only patients who underwent all three modalities may introduce selection bias. Although the T-score is widely used, it does not capture all biomechanical or metabolic contributors to fracture risk. Subgroup analyses were descriptive, and some strata—particularly high-risk osteoporosis—had small sample sizes, which restricts the reliability of comparisons. Furthermore, MRI availability and workflow considerations vary across institutions, potentially limiting applicability in resource-constrained settings. Finally, the absence of longitudinal follow-up prevented the evaluation of the prognostic impact of fractures detected only by STIR MRI.

Future multicenter prospective studies with larger cohorts and standardized imaging protocols are needed to validate these findings, refine T-score-based triage strategies, and assess cost-effectiveness in routine practice.

## 5. Conclusions

STIR MRI consistently outperformed CT in vertebral fracture detection across all bone mineral density categories, including patients with normal and osteopenic T-scores. While osteoporosis (T-score ≤ −2.5) warrants STIR MRI almost by default when there is clinical suspicion, our findings demonstrate that patients with higher T-scores may also harbor occult fractures not visible on CT.

Importantly, correlation analyses did not identify statistically significant thresholds, indicating that the T-score should not be used as a strict decision cut-off. Instead, it should be considered a supportive parameter that complements clinical findings and imaging results. Integrating T-score into imaging triage may help guide timely escalation from CT to STIR MRI, improve diagnostic accuracy, and reduce underdiagnosis across the bone density spectrum.

Future multicenter prospective studies are required to validate standardized thresholds, assess cost-effectiveness, and determine how T-score-based triage can be incorporated into routine clinical practice.

## Figures and Tables

**Table 1:** Paired CT vs. STIR MRI by T-score category: n, median MRI–CT difference (MRI−CT), Wilcoxon *p*, and Holm–Bonferroni–adjusted *p*; positive differences indicate more fractures on MRI.

T-Score Category	n Patients	Median MRI–CT Difference	Wilcoxon *p*-Value	Holm–Bonferroni Adjusted *p*-Value
Normal	54	0	0.0007	0.0028
Osteopenia	91	0	<0.000001	<0.000004
Osteoporosis	66	0	0.00004	0.00016
High-risk Osteoporosis	21	1	0.0046	0.0184

**Table 2:** This table illustrates the Age-Stratified T-Score and Risk Distribution for osteoporosis and by T-score Category in the Study Cohort.

Age Group	Total Patients	Low Risk		Total n (%)	Higher Risk		Total n (%)
		Normal n (%)	Osteopenia n (%)		Osteoporosis n (%)	High-Risk Osteoporosis n (%)	
50–59	31	10 (32.3)	13 (42.0)	26 (74,3)	7 (22.6)	1 (3.2)	8 (25.8)
60–69	46	15 (32.6)	21 (45.7)	36 (78.3)	7 (15.2)	3 (6.5)	10 (21.7)
70–79	80	20 (25.0)	30 (37.5)	50 (62.5)	19 (23.8)	11 (13.8)	30 (37.5)
80–89	50	11 (22.0)	21 (42)	33 (64)	12 (24.0)	6 (12.0)	18 (36)
90–99	7	0	6 (85.7)	6 (85.7)	0	1 (14.3)	1 (14.3)
Total	214	56	91		45	22	

## Data Availability

All data generated or analyzed during this study are included in this published article.
